# The Genotypic False Positive Rate Determined by V3 Population Sequencing Can Predict the Burden of HIV-1 CXCR4-using Species Detected by Pyrosequencing

**DOI:** 10.1371/journal.pone.0053603

**Published:** 2013-01-14

**Authors:** Valentina Svicher, Valeria Cento, Gabriella Rozera, Isabella Abbate, Maria Mercedes Santoro, Daniele Armenia, Lavinia Fabeni, Alessandro Bruselles, Alessandra Latini, Guido Palamara, Valeria Micheli, Giuliano Rizzardini, Caterina Gori, Federica Forbici, Giuseppe Ippolito, Massimo Andreoni, Andrea Antinori, Francesca Ceccherini-Silberstein, Maria Rosaria Capobianchi, Carlo Federico Perno

**Affiliations:** 1 Department of Experimental Medicine and Surgery, University of “Tor Vergata” Rome, Italy; 2 I.N.M.I. “L. Spallanzani”, Rome, Italy; 3 “San Gallicano” Hospital, Rome, Italy; 4 “L. Sacco” Hospital, Milan, Italy; 5 University Hospital “Tor Vergata”, Rome, Italy; British Columbia Centre for Excellence in HIV/AIDS, Canada

## Abstract

**Objective:**

The false-positive rate (FPR) is a percentage-score provided by Geno2Pheno-algorithm indicating the likelihood that a V3-sequence is falsely predicted as CXCR4-using. We evaluated the correlation between FPR obtained by V3 population-sequencing and the burden of CXCR4-using variants detected by V3 ultra-deep sequencing (UDPS) and Enhanced-Sensitivity Trofile assay (ESTA).

**Methods:**

54 HIV-1 B-subtype infected-patients (all maraviroc-naïve), with viremia >10,000copies/ml, were analyzed. HIV-tropism was assessed by V3 population-sequencing, UDPS (considering variants with >0.5% prevalence), and ESTA.

**Results:**

By UDPS, CCR5-using variants were detected in 53/54 patients, irrespective of FPR values, and their intra-patient prevalence progressively increased by increasing the FPR obtained by V3 population-sequencing (rho = 0.75, p = 5.0e-8). Conversely, the intra-patient prevalence of CXCR4-using variants in the 54 patients analyzed progressively decreased by increasing the FPR (rho = −0.61; p = 9.3e-6). Indeed, no CXCR4-using variants were detected in 13/13 patients with FPR>60. They were present in 7/18 (38.8%) patients with FPR 20–60 (intra-patient prevalence range: 2.1%–18.4%), in 5/7 (71.4%) with FPR 10–20, in 4/6 (66.7%) with FPR 5–10, and in 10/10(100%) with FPR<5 (intra-patient prevalence range: 12.1%–98.1%).

**Conclusions:**

FPR by V3 population-sequencing can predict the burden of CXCR4-using variants. This information can be used to optimize the management of tropism determination in clinical practice. Due to its low cost and short turnaround time, V3 population-sequencing may represent the most feasible test for HIV-1 tropism determination. More sensitive methodologies (as UDPS) might be useful when V3 population-sequencing provides a FPR >20 (particularly in the range 20–60), allowing a more careful identification of patients harboring CXCR4-using variants.

## Introduction

Human immunodeficiency virus type 1 (HIV-1) entry into host cells requires coordinated interactions of the envelope glycoprotein gp120 with the CD4 receptor and with one of the chemokine receptors, CCR5 or CXCR4. Pure CCR5-tropic and pure CXCR4-tropic virus use only the CCR5 and CXCR4 co-receptors to enter target-cells, respectively, while dual-tropic virus can use both co-receptors [1].

The impact of HIV-1 co-receptor usage has been correlated with the rate of disease progression in HIV-1 infected individuals [2]–[4]. Determining HIV-1 co-receptor usage is also critical since the CCR5 co-receptor has become the target of a new class of anti-HIV-1 drugs that specifically inhibit the entry of CCR5-tropic HIV-1 strains into the target cells by allosteric inhibition of the CCR5 co-receptor [5]. Maraviroc is the first approved CCR5 antagonist, that entered clinical practice in 2007. Since then, assessment of HIV-1 co-receptor usage is mandatory for the clinical use of this drug (http://www.aidsinfo.nih.gov/ContentFiles/AdultandAdolescentGL.pdf) [6].

HI V-1 co-receptor usage can be assessed with either phenotypic or genotypic approaches. The commercial Trofile assay (Monogram Biosciences, San Francisco, California, USA), and its newer version the enhanced sensitivity Trofile assay (ESTA) (with a nominal lower limit of sensitivity of 0.3% for detecting CXCR4-using virus within clonal mixture) have been so far the most widely applied phenotypic test. Due to logistical and financial limitations of Trofile assays, different genotypic assays have been developed (http://www.aidsinfo.nih.gov/ContentFiles/Adultand AdolescentGL.pdf) [6]. They are based on the amplification and sequencing of the patient’s derived HIV-1 gp120 V3 domain, which is the major determinant for co-receptor binding [7]–[9].

Two approaches have been used for V3 sequencing: V3 population sequencing and V3 ultra-deep pyrosequencing (UDPS). V3 population sequencing is currently used in routine clinical practice especially in Europe, while UDPS is mainly used for research purposes [6], [10]–[21]. In comparison to V3 population sequencing, UDPS can capture a detailed cross-section of co-receptor use across a patient’s viral population and quantify the prevalence of CXCR4-using variants within the patient. The genetic information contained in the V3 sequence (generated by either V3 population- or ultra-deep sequencing) is then used to infer HIV-1 tropism by using web-based bioinformatic interpretation algorithms. Among them, Geno2pheno (http://coreceptor.bioinf.mpiinf.mpg.de/) is so far the most commonly used interpretation algorithm in clinical practice in Europe [6]. For the tropism prediction, Geno2Pheno provides a score, called false-positive rate (FPR). FPR is a percentage score (range 0–100) indicating the likelihood that a V3 sequence is falsely predicted as CXCR4-using. Thus, a viral sequence with high FPR has a high probability to be CCR5-using. Although several studies have investigated the performances of genotypic tropism testing (based on V3 population sequencing) in comparison with phenotypic testing [16], [18], [19], [22], none of them has investigated the potential correlation between the FPR and the burden of CXCR4- or CCR5-using species circulating in a patient.

In this light, this study is aimed at: i) investigating the correlation between FPR by V3 population sequencing and the burden of X4-species, detected by UDPS; ii) analyzing the correlation between quasispecies diversity and frequency of CXCR4-using variants.

## Methods

### Patients

Stored plasma samples derived by clinical routine assessment of HIV-1 resistance from fifty-four HIV-1 infected patients were retrospectively retrieved and included in the analysis. Ethic approval was deemed unnecessary because, under Italian law, biomedical research is subjected to previous approval by ethics committes only in the hypothesis of clinical trials on medicinal products for clinical use (art. 6 and art. 9, leg. decree 211/2003). The research also was conducted on RNA samples and data previously anonymized, according to the requirements set by Italian Data Protection Code (leg. decree 196/2003). All of the selected specimen had a viral load >10,000 copies/ml at the time of sampling, and they were all infected by HIV-1 subtype B, as determined by phylogenetic analysis of *pol* sequences, and confirmed by V3 analysis [19]. For each specimen, HIV-1 tropism was assessed by V3 population-sequencing (based on a single PCR) and V3 ultra-deep sequencing (based on 4 PCR replicates). For 44 out 54 samples, viral tropism was also determined phenotypically by ESTA.

### V3 Population Sequencing

The protocol for V3 population sequencing based on single round of amplification has been generated and optimized as previously described [18], [19]. A detailed description of this protocol is reported in SI text (S1).

### V3 Ultradeep Pyrosequencing

UDPS was carried out with the 454 Life Sciences platform (GS-FLX; Roche Applied Science) as described in [10], [11], [17], on plasma samples from all the 54 enrolled patients. Nucleic acid extraction, quantification of the templates actually undergoing UDPS and V3-specific reverse transcription PCR were performed as described in [17]. Unique in-house designed stretches of eight nucleotides (multiplex identifiers) were used to tag each sample. To maximize the genetic heterogeneity of viral population present in 1 ml of plasma and thus to ensure a good sampling of the viral population, amplicons from at least 4 replicate PCR reactions were pooled for each sample. To minimize most of the procedural/experimental errors, due to error rate of the high-fidelity polymerase and the high-throughput pyrosequencing platform, a correction pipeline was adopted as previously described in [12], [17]. In particular, after translation of nucleotide sequences, only the coding ones, having at least one forward and one reverse sequence, have been analysed.

To estimate the UDPS error rate, a plasmid clone containing the region of interest was sequenced in parallel with the Sanger method [9], [14]. Any nucleotide differences between the two methods were considered to be GS-FLX sequencing errors. Within the env region, the crude error rate was 0.43%, reduced to 0.058% after the application of the correction pipeline (0.043% for non-homopolymeric regions and 0.11% for homopolymeric regions). Taking into account the estimated error rate for the high-fidelity polymerase used to obtain the amplicons (∼1×10−6 mutations/bp per duplication), mutation frequencies at each nucleotide site, exceeding by at least eight times the corrected error rate, were considered to reflect true variability and not procedural/experimental errors by our in-house developed correction pipeline. Considering the number of viral templates actually undergoing UDPS and the corrected error rate, the threshold of sensitivity was set to 0.5%.

### Genotypic Prediction of Viral Tropism

HIV-1 co-receptor usage was inferred from the V3 nucleotide sequence by using the geno2pheno algorithm available at the following website http://coreceptor.bioinf.mpi-inf.mpg.de/. HIV-1 co-receptor usage of V3-sequences, obtained by both population and ultra-deep sequencing, was inferred by using the clonal version of geno2pheno set at FPR of 5.75. This cut-off, used in all the analyses carried out in this study, was chosen since it has been shown to be a good predictor of virological response to a maraviroc-containing regimen in both multi-experienced and drug-naïve patients [6], [14], [20]. In addition, to estimate the concordance, sensitivity and specificity of tropism prediction by UDPS using ESTA as reference, a FPR of 5.75 and 10 was used.

### Heterogeneity Parameters Calculation

The amino acid UDPS sequences resulting from the correction pipeline were analyzed to assess diversity and quasispecies complexity. To assess diversity, the mean genetic distance of amino acid sequences was calculated by PROTDIST using Jones-Taylor-Thornton matrix and with an in-house written code. Quasispecies complexity was calculated using normalized Shannon entropy (Sn = -Σ(pi ln pi)/ln N), where pi was the frequency of each distinct nucleotide sequence and N was the total number of sequences analyzed.

### Statistical Analysis

Data were analyzed using the statistical software package SPSS (SPSS Inc., Chicago, IL). In particular, the correlation between the prevalence of X4 and R5 variants and the values of FPR at V3 population sequencing was assessed by Spearman’s rank correlation coefficient. P-values less than 0.05 were considered statistically significant.

## Results

### 

#### Patients’ characteristics

This study included 54 HIV-1 (all B subtype) infected patients: 15 HAART-naïve and 39 HAART-experienced (Table 1). All patients were naïve to maraviroc and investigational CCR5 antagonists, and 3 have experienced the fusion inhibitor enfuvirtide in their therapeutic history. At the time of sample collection, median (IQR) viremia was 4.9 (4.5–5.3) log_10_ HIV-1 RNA copies/ml, and median (IQR) CD4 cell count was 254 (107–349) cells/ul (Table 1).

**Table 1:** Demographic characteristics of the study population.

Characteristic		
**Patients, N**		54
**Male, N(%)**		45 (81.8)
**Age (years), Median (IQR)**		40.0 (35.0–44.1)
**Risk Factor, N(%)**	HE	1 (1.8)
	MSM	13 (23.6)
	IDU	9 (16.4)
	ND	26 (47.2)
**Time from HIV-1 diagnosis (years), Median (IQR)**		12.3 (2.3–17.3)
**Log HIV-1 RNA (IU/ml), Median (IQR)**		4.9 (4.5–5.3)
**CD4 T-cell count (cell/ul), Median (IQR)**		254 (107–349)
**Therapy status**		
Drug-Naïve, N (%)		15 (27.8)
Drug-Experienced, N (%)		39 (72.2)
HAART Length (years), Median (IQR)		11.8 (9.7–15.3)

IQR, interquartile range; HE, heterosexual; MSM, men-who-have-sex-with-men; IDU, injection drug user; ND, not determined; IU, international units.

### Co-receptor Usage Prediction by Viral Quasispecies

V3 population sequencing (set at a FPR of 5.75) identified 10/54 (18.5%) samples as CXCR4-using (4/15 drug-naive and 6/39 in drug-experienced patients, P = 0.339), and had 76.6% concordance with ESTA, in line with other previous studies [13], [16], [18], [22]. Similarly, UDPS (set at a FPR of 5.75) showed a 76.1% concordance with ESTA, with a sensitivity and specificity of 78.9% and 71.4%, respectively. Conversely, using a FPR of 10, the sensitivity and specificity of UDPS raised to 94.7% and dropped to 50%, respectively.

**Figure 1:**
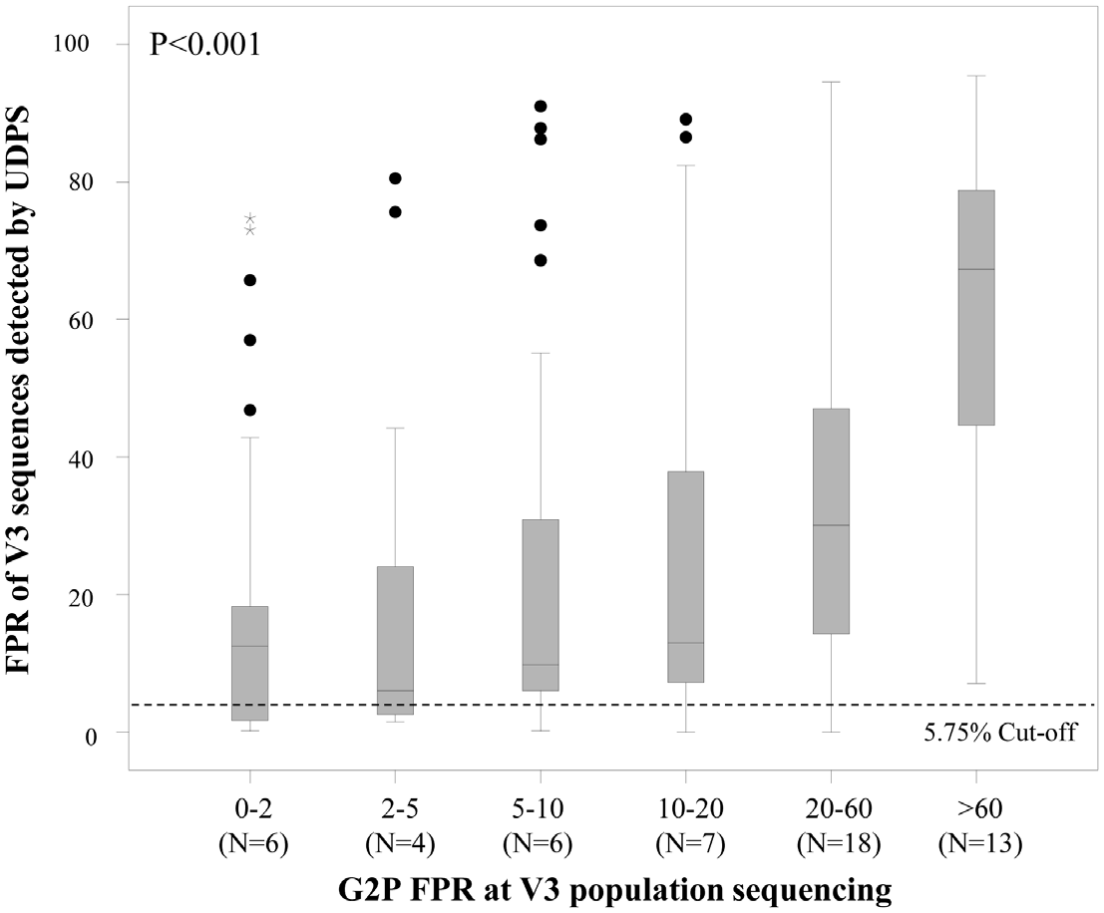
Box plot reporting the distribution of FPR values of V3 sequences obtained by UDPS, sorted according to the FPR value at population sequencing. The medians, interquartile ranges, upper and lower whiskers, and outlier values are shown. P-value was calculated through Kruskal-Wallis Test.

We observed that the FPR obtained by V3 population sequencing was directly correlated with the median FPR of V3 sequences detected by UDPS (p<0.001) (Fig.1), thus suggesting that the CCR5 usage of the entire viral population progressively increases with the FPR at V3 population sequencing.

Overall, UDPS detected CXCR4-using variants (with at least a prevalence >0.5%) in 26/54 (48.1%) patients, while 28/54 (51.9%) showed 100% CCR5-using viruses in their quasispecies population. The intra-patient prevalence of X4 variants in these 26 patients showed a wide range from 0.6% to 100% of the viral population (median [IQR]:27.4% [4.8%–81.4%]), corresponding to an X4-load (based on total viral load) ranging from 186 copies/ml to 129,107 copies/ml (median [IQR]: 14838 [3715–43253] copies/ml). Only 1 patient showed 100% CXCR4-using variants with a FPR range of 1.5–5.3 by UDPS, and a FPR value of 2.7 at population sequencing.

In drug-naïve patients, CXCR4-using variants were detected by UDPS in 8/15 (53.3%) of them, with intra-patient prevalence ranging from 2.3% to 99.1% (median [IQR]: 27.4% [4.2%–43.7%]), and with a median (IQR) X4-load of 21,972 (13,511–90,210) copies/ml. Among them, 4 have an intra-patient prevalence of X4-variants <20% that is generally the limit of detection by population sequencing.

Similarly, CXCR4-using variants were detected in 18/39 (46.1%) drug-experienced patients, with a median [IQR] X4-load of 8,754 [3086–29,621] copies/ml. Their intra patient prevalence was 47.7% [7.6%–92%], higher than that observed in drug-naive patients (27.4% [4.2%–43.7%]). The difference in the prevalence of CXCR4-using variants in drug-naive versus drug-experienced patients was not statistically significant (53.3% versus 46.1%, P = 0.636).

### Correlation between the FPR by V3 Population Sequencing and the Amount of R5 and X4 Species Detected by V3 Ultra-deep Sequencing

**Figure 2:**
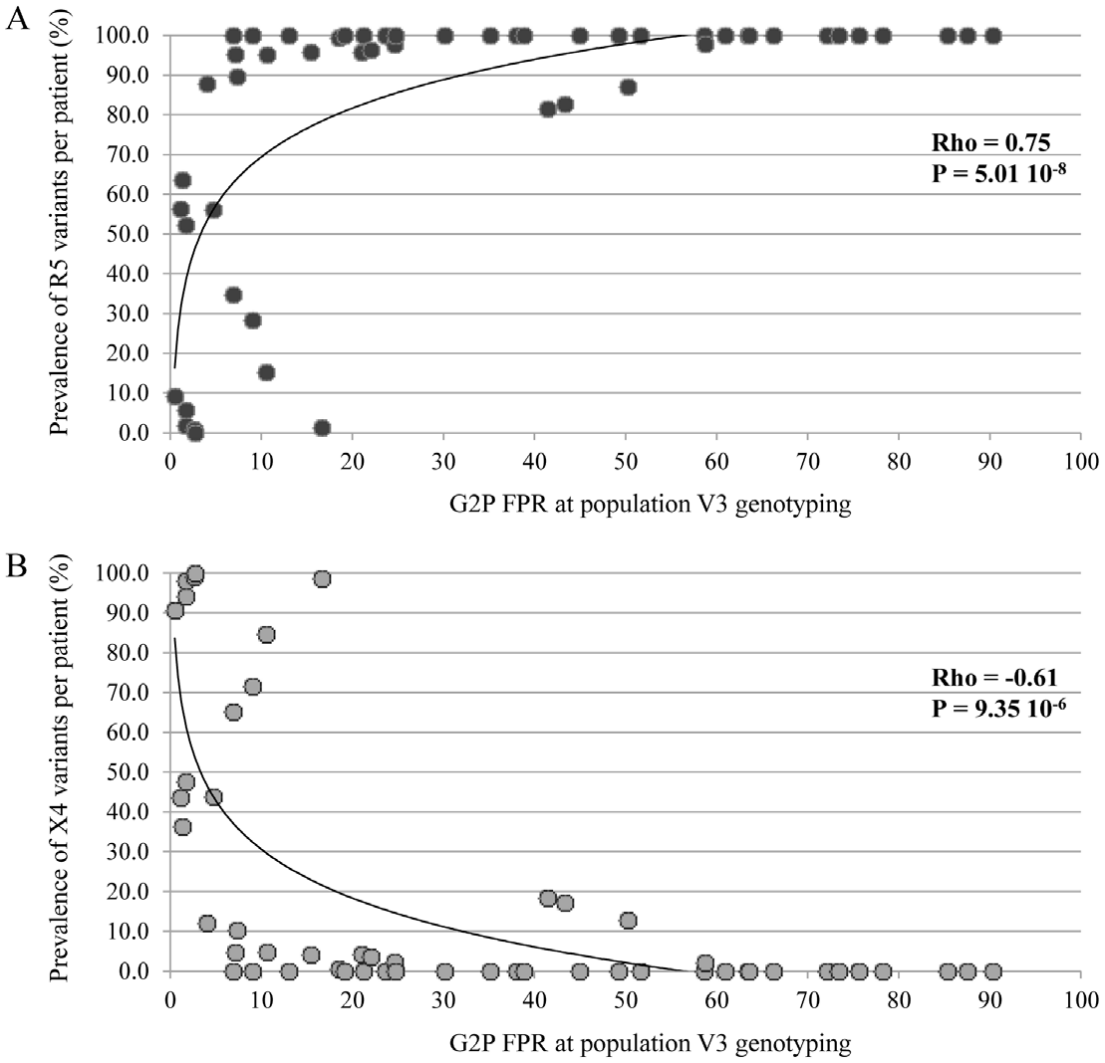
The graphs report the proportion of R5 (A) and X4 (B) variants per patient according to the values of FPR at population V3 sequencing. Distribution of R5 and X4 variants in relationship to the False Positive Rate (FPR) detected by population V3 sequencing. The graphs report the proportion of R5 (A) and X4 (B) variants per patient according to the values of FPR at population V3 sequencing. P-values were calculated by Spearman test. A FPR of 5.75 has been used as cut-off to infer HIV-1 co-receptor usage.

A next step of this study was to evaluate the correlation between the FPR detected by V3 population sequencing and the burden of CXCR4-using species detected by UDPS. In this analysis, at least 1 CCR5-using variant was detected in 53 out 54 patients, irrespective of FPR values obtained by population V3 sequencing. Their intra-patient prevalence progressively increased by increasing the FPR (rho = 0.75, p = 5.0e-8) (Fig.2), while intra-patient prevalence of X4 variants progressively decreased by increasing the FPR (rho =  −0.61; p = 9.3e-6) (Fig.2).

**Figure 3:**
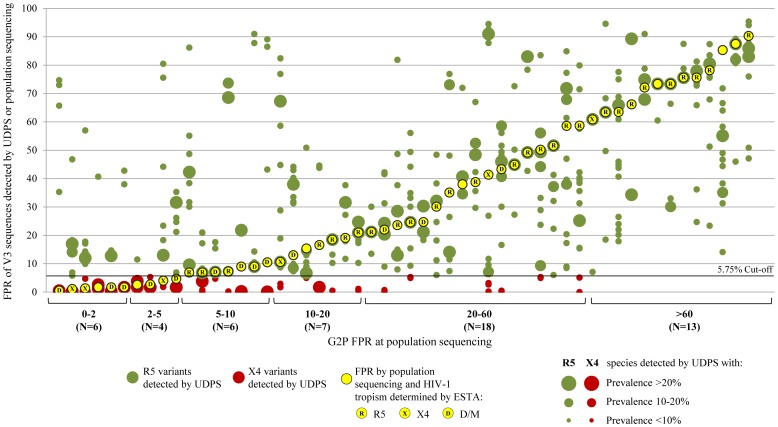
The graph reports the distribution of FPR values of all the V3 variants detected by UDPS in each patient according to FPR ranges at population V3 sequencing. The relative dimension of green and red dots represents the prevalence of R5 and X4 species detected by UDPS. Yellow dots represent the FPR determined by population sequencing and letters within dots indicate the phenotypic tropism determined by ESTA (R =  pure CCR5 tropism, X = pure CXCR4 tropism, D = dual/mixed tropism. For blank yellow dots, ESTA result was not available. A FPR of 5.75 has been used as cut-off to infer HIV-1 co-receptor usage of V3 sequences obtained by both V3 population and ultra-deep sequencing.

In detail, in 13/13 (100%) patients with FPR >60 by V3 population sequencing, only CCR5-using variants were detected by UDPS (FPR range: 7.1–95.4), without any X4 variants (detection limit of 0.5% prevalence, FPR<5.75) (Table 2, Fig.3). Among the 10 patients with ESTA available, 9 were also with phenotypic tropism R5; for the remaining patient, both V3 population sequencing and UDPS reported R5-tropism, while ESTA reported an X4-tropism.

**Table 2:** UDPS species prevalence according to G2P FPR at population V3 genotyping.

FPR at V3 Populationsequencing	N ofPatients	X4 prevalence (%) range^a^	R5 prevalence(%) range^a^	N° (%) of patients according to the following ranges of X4 species^b^:					N° (%) of patients according to the following ranges of R5 species^b^:					
				≤2	2.1–10	10.1–50	50.1–90	>90.1	≤2	2.1–10	10.1–50	50.1–90	>90.1	
≤2	6	36.4–98.1	1.9–63.6	0 (0.0)	0 (0.0)	3 (50.0)	0 (0.0)	3 (50.0)	1 (16.7)	2 (33.3)	0 (0.0)	3 (50.0)	0 (0.0)	
2–5	4	12.1–100	0.0–87.9	0 (0.0)	0 (0.0)	1 (25.0)	1 (25.0)	2 (50.0)^c^	1 (25.0)	0 (0.0)	0 (0.0)	2 (50.0)	0 (0.0)	
5–10	6	0.0–71.6	28.4–100	0 (0.0)	1 (16.7)	1 (16.7)	2 (33.3)	0 (0.0)	0 (0.0)	0 (0.0)	2 (33.3)	1 (16.7)	3 (50.0)	
10–20	7	0.0–98.7	1.3–100	1 (14.3)	2 (28.6)	0 (0.0)	1 (14.3)	1(14.3)	1 (14.3)	0 (0.0)	1 (14.3)	0 (0.0)	5 (71.4)	
20–60	18	0.0–18.4	81.6–100	0 (0.0)	4 (22.2)	3 (16.7)	0 (0.0)	0 (0.0)	0 (0.0)	0 (0.0)	0 (0.0)	3 (16.7)	15(83.3)	
≥60	13	0.0	100	0 (0.0)	0 (0.0)	0 (0.0)	0 (0.0)	0 (0.0)	0 (0.0)	0 (0.0)	0 (0.0)	0 (0.0)	13 (100)	

a The column reports the range of prevalence for CXCR4-using or CCR5-using strains determined by UDPS in patients stratified according to the FPR values obtained by V3 population sequencing.

b The ranges are referred to the intra-patient prevalence of X4- and R5-species by UDPS.

c The intra-patient prevalence of X4-variants is 99.1% and 100%, respectively.

Abbreviations: UDPS, ultra-deep sequencing; G2P, Geno2Pheno; FPR, false positive rate.

Many patients (11/18 [61.1%]) with FPR ranging from 20 to 60 by V3 population sequencing were infected only by CCR5-using variants (Table 2, Fig.3). In the remaining 7 patients, minority CXCR4-using variants were detected, with an intra-patient prevalence ranging from 2.1% to 18.4% (median [IQR] prevalence: 4.3% [3.0% −15.1%]) corresponding to an X4 load ranging from 186 copies/ml to 26,026 (median [IQR] prevalence: 1,336 [426–7,322] copies/ml) (Table 2, Fig.3).

The proportion of patients with CXCR4-using variants increased for values of FPR <20 by V3 population sequencing (Fig.3). In particular, they were present in 5/7 (71.4%) with FPR 10–20, in 4/6 (66.7%) with FPR 5–10, in 10/10 (100%) with FPR <5 (Table 2, Fig.3). In this latter group of patients, X4 species showed intra-patient prevalence ranging from 12.1% to 100% of the entire viral population, with a median (IQR) X4-load of 51,483 (14,161–81,762) copies/ml.

### X4 Variants and Intra-patient Quasispecies Diversity Relationship

**Figure 4:**
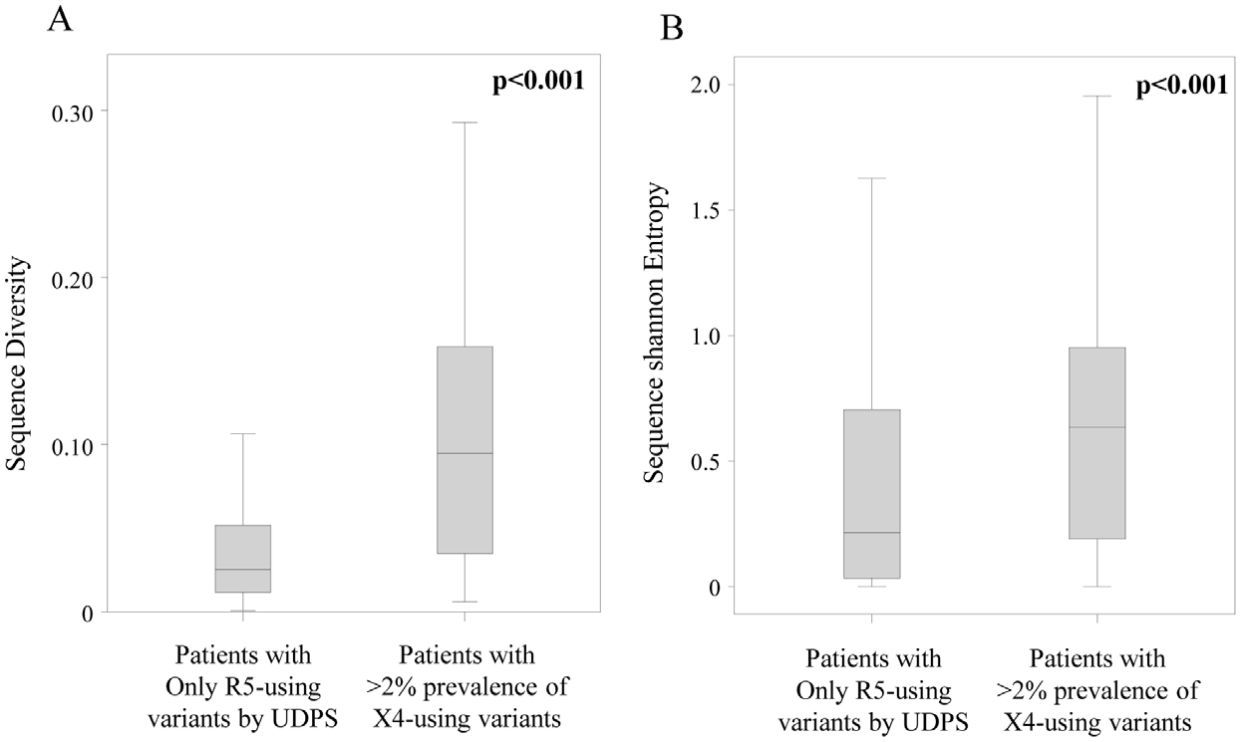
Quasispecies heterogeneity. The box plots represent the diversity of amino acid sequences (A), and Shannon entropy (B) among patients with X4 species detected by UDPS at a prevalence lower or higher than 1%. P-values were calculated by Mann-Whitney Test.

The quasispecies diversity and variability (represented by Shannon Entropy, see materials and methods section) at a protein level ranged from 0.012 subs/site to 0.222 subs/site and from 0.02 to 1.18, respectively, and significantly correlated with intra-patient prevalence of CXCR4-using variants (Rho = 3.76, P = 1.7e-4 for diversity and Rho = 4.76, P = 2.0e-6 for Shannon Entropy, by Spearman Test) (data not shown). In particular, the median [IQR] diversity of amino acid sequences was higher in patients with X4 variants >2% (0.09 [0.03–0.16] subs/site) than in patients with only CCR5-using variants (0.03 [0.01–0.05]) (P<0.001 by Mann-Whitney test) (Fig. 4, panel A). Similarly, the median [IQR] Shannon Entropy was 0.634 [0.19–0.95] in patients with X4 variants >2% and 0.22 [0.03–0.70] in patients with only CCR5-using variants (P<0.001 by Mann-Whitney test) (Fig. 4, panel B). This result was confirmed by stratifying patients according to ESTA. Indeed, both the genetic distance and the Shannon entropy was higher in samples classified by ESTA as non-R5-using than samples classified as R5-using (0.06 [0.03–0.16] in non-R5-using versus 0.03 [0.01–0.09] in R5-using, P = 0.050; 0.63 [0.23–1.00] in non-R5-using versus 0.45 (0.09–0.78) in R5-using, P = 0.015, respectively). Thus, overall results supports a greater quasispecies diversity in patients carrying X4-variants.

## Discussion

This study highlights a direct correlation between the FPR detected by V3 population sequencing and the burden of CXCR4-using species detected by UDPS in HIV-1 B-subtype infected patients.

In particular, no CXCR4-using variants were detected in patients with FPR >60 by V3 population sequencing. These results were supported in an independent dataset of 15 HIV-1 infected patients tested for HIV-1 tropism by V3 ultra-deep sequencing (454 GS-Junior). In this dataset, none of the 3 patients with FPR>60 had X4 variants (0.1% cut-off) (Ceccherini-Silberstein et al., personal communication). These results can also explain a recent study aimed at determining the prevalence and the correlates of co-receptor switch in antiretroviral-naïve patients [20]. The authors found that the FPR, obtained by V3 population sequencing at baseline, was the only variable associated with co-receptor switch in the observation period of 2 years. In particular, no switches from R5-using virus to X4-virus were observed in patients with FPR>50 [20].

For 1 patient with FPR of 60.9 by V3 population sequencing, exceptionally, the ESTA result reported an X4-tropism while UDPS reported only the presence of R5-using species. Discordances between genotypic and phenotypic tropism testing have been previously described, and can be explained by the existence of additional positions in the env gp160, beyond those within the V3 loop, which may influence viral tropism [23]–[27]. Moreover, due to the laborious ESTA procedure, we cannot exclude that such genotypic/phenotypic discordance may be due to technical issues.

Interestingly, in our study, the intra-patient prevalence of CXCR4-using variants by UDPS progressively decreased by increasing the FPR obtained by V3 population sequencing. In particular, CXCR4-using variants were observed in 38.9% (7/18) of patients with FPR ranging from 20 to 60 (X4 prevalence: 2.1%–18.4%), in 75% (9/12) patients with FPR ranging from 5 to 20 (range X4 prevalence 0.6%–98.7%), and in 100% (10/10) patients with FPR<5 (range X4 prevalence 12.1%–100%). The presence of CXCR4-using variants in almost all patients with FPR <20 by population V3 sequencing is in line with the current guidelines [6] recommending a FPR of 20% as cut-off for the identification of patients candidate to maraviroc treatment when genotypic testing is based on a single round of PCR amplification.

Furthermore, for the specific set of patients with FPR ranging from 20 to 60, V3 population sequencing (based on single amplification) may also not be sufficient for proper determination of HIV-1 tropism, and thus, more sensitive methodologies, such as V3 UDPS or the phenotypic ESTA, might be used to identify more precisely patients candidate to maraviroc treatment. This is important since analyses from the MERIT and MOTIVATE trials have recently shown that the presence of as little as 2% of non-R5 viruses is independently associated with an increased risk of virological failure to maraviroc-containing regimens [17], [20], [28].

In particular, V3 UDPS has been shown to be highly predictive of clinical outcome to CCR5 antagonist in retrospective analyses of large clinical studies [17]. However, it can be achieved so far only in specialized settings (mainly at specific academic or commercial service units), and, since it is expensive and requires much computing capacity and interpretation expertise, its use in current routine clinical practice could be limited. Nevertheless, our results (even if based on a small number of patients) may suggest a guided-use of V3 UDPS, especially for patients with FPR ranging from 20 to 60. This could contribute to rationalize the use of this methodology in clinical practice.

For all these reasons, genotypic testing based on V3 population sequencing still remains the preferred test for tropism determination in several clinical settings. In this light, this study contributes to further support the use of genotypic testing as valid testing for tropism determination in line with the recommendation of recent guidelines on clinical management of HIV-1 tropism testing [6].

Finally, it is intriguing that, in line with previous results [12], intra-patient X4 frequencies were always positively correlated with parameters of quasispecies heterogeneity. This finding may suggest either a possible evolutionary pathway, during which heterogeneity accumulation is necessary to give rise to X4 variants, or, otherwise, that X4 variants are intrinsically more heterogeneous. Studies on longitudinal samples are needed in order to confirm this hypothesis.

In conclusion, this study shows that the FPR determined by V3 population sequencing can predict the burden of CXCR4-using variants in the infecting viral quasispecies, and suggests to use the FPR score with more attention before CCR5 antagonist prescription. Due to its low cost and short turnaround time, V3 population sequencing may represent the most feasible test for HIV-1 tropism determination. More sensitive methodologies might be useful when V3 population sequencing provides a FPR >20 and particularly in the range from 20 to 60, allowing a better identification of patients harboring CXCR4-using variants.

## Supporting Information

Text S1 V3 population-sequencing.
(DOC)

## References

[1] Berger EA, Doms RW, Fenyo EM, Korber BT, Littman DR, et al.. (1998) A new classification for HIV-1. Nature 391: 240. 10.1038/34571 [doi]. 10.1038/34571 9440686

[2] Regoes RR, Bonhoeffer S (2005) The HIV coreceptor switch: a population dynamical perspective. Trends Microbiol 13: 269–277. S0966-842X(05)00110-1 [pii];10.1016/j.tim.2005.04.005 [doi]. 10.1016/j.tim.2005.04.005 15936659

[3] Waters L, Mandalia S, Randell P, Wildfire A, Gazzard B, et al.. (2008) The impact of HIV tropism on decreases in CD4 cell count, clinical progression, and subsequent response to a first antiretroviral therapy regimen. Clin Infect Dis 46: 1617–1623. 10.1086/587660 [doi]. 10.1086/587660 18419499

[4] Raymond S, Delobel P, Mavigner M, Cazabat M, Encinas S, et al. (2010) CXCR4-using viruses in plasma and peripheral blood mononuclear cells during primary HIV-1 infection and impact on disease progression. AIDS 24: 2305–2312. 10.1097/QAD.0b013e32833e50bb [doi]. 10.1097/QAD.0b013e32833e50bb 20808203

[5] Macarthur RD, Novak RM (2008) Reviews of anti-infective agents: maraviroc: the first of a new class of antiretroviral agents. Clin Infect Dis 47: 236–241. 10.1086/589289 [doi]. 10.1086/589289 18532888

[6] Vandekerckhove LP, Wensing AM, Kaiser R, Brun-Vezinet F, Clotet B, et al.. (2011) European guidelines on the clinical management of HIV-1 tropism testing. Lancet Infect Dis 11: 394–407. S1473-3099(10)70319-4 [pii];10.1016/S1473-3099(10)70319-4 [doi]. 10.1016/S1473-3099(10)70319-4 21429803

[7] Cardozo T, Kimura T, Philpott S, Weiser B, Burger H, et al.. (2007) Structural basis for coreceptor selectivity by the HIV type 1 V3 loop. AIDS Res Hum Retroviruses 23: 415–426. 10.1089/aid.2006.0130 [doi]. 10.1089/aid.2006.0130 17411375

[8] Hoffman TL, Doms RW (1999) HIV-1 envelope determinants for cell tropism and chemokine receptor use. Mol Membr Biol 16: 57–65. 10332738 10.1080/096876899294760

[9] Nolan KM, Jordan AP, Hoxie JA (2008) Effects of partial deletions within the human immunodeficiency virus type 1 V3 loop on coreceptor tropism and sensitivity to entry inhibitors. J Virol 82: 664–673. JVI.01793-07 [pii];10.1128/JVI.01793-07 [doi]. 10.1128/JVI.01793-07 PMC2224606 17977968

[10] Abbate I, Vlassi C, Rozera G, Bruselles A, Bartolini B, et al.. (2011) Detection of quasispecies variants predicted to use CXCR4 by ultra-deep pyrosequencing during early HIV infection. AIDS 25: 611–617. 10.1097/QAD.0b013e328343489e [doi]. 10.1097/QAD.0b013e328343489e 21160417

[11] Abbate I, Rozera G, Giombini E, D’Offizi G, Nicastri E, et al.. (2011) Deep Sequencing of Plasma and Proviral HIV-1 to Establish Coreceptor Usage: What Is the Clinical Impact of the Quasispecies Distribution? J Infect Dis 204: 971–973. jir427 [pii];10.1093/infdis/jir427 [doi]. 10.1093/infdis/jir427 21849294

[12] Abbate I, Rozera G, Tommasi C, Bruselles A, Bartolini B, et al.. (2011) Analysis of co-receptor usage of circulating viral and proviral HIV genome quasispecies by ultra-deep pyrosequencing in patients who are candidates for CCR5 antagonist treatment. Clin Microbiol Infect 17: 725–731. CLM3350 [pii];10.1111/j.1469-0691.2010.03350.x [doi]. 10.1111/j.1469-0691.2010.03350.x 20731681

[13] Chueca N, Garrido C, Alvarez M, Poveda E, de Dios LJ, et al.. (2009) Improvement in the determination of HIV-1 tropism using the V3 gene sequence and a combination of bioinformatic tools. J Med Virol 81: 763–767. 10.1002/jmv.21425 [doi]. 10.1002/jmv.21425 19319937

[14] Harrigan R, Zhong X, Lewis M, Dong W, Knapp D, et al (2010) The influence of PCR amplification variation on the ability of population-based PCR to detect non-R5 HIV. Rewievs in Antiviral Therapy & Infectious Diseases, Abstract Book of 8th European HIV Drug Resistance Workshop 1: 36–37.

[15] McGovern RA, Harrigan PR, Swenson LC (2010) Genotypic inference of HIV-1 tropism using population-based sequencing of V3. J Vis Exp. 2531 [pii];10.3791/2531 [doi]. 10.3791/2531 PMC3159650 21248683

[16] Poveda E, Seclen E, Gonzalez MM, Garcia F, Chueca N, et al.. (2009) Design and validation of new genotypic tools for easy and reliable estimation of HIV tropism before using CCR5 antagonists. J Antimicrob Chemother 63: 1006–1010. dkp063 [pii];10.1093/jac/dkp063 [doi]. 10.1093/jac/dkp063 19261623

[17] Rozera G, Abbate I, Bruselles A, Vlassi C, D’Offizi G, et al.. (2009) Massively parallel pyrosequencing highlights minority variants in the HIV-1 env quasispecies deriving from lymphomonocyte sub-populations. Retrovirology 6: 15. 1742-4690-6-15 [pii];10.1186/1742-4690-6-15 [doi]. 10.1186/1742-4690-6-15 PMC2660291 19216757

[18] Svicher V, D’Arrigo R, Alteri C, Andreoni M, Angarano G, et al (2010) Performance of genotypic tropism testing in clinical practice using the enhanced sensitivity version of Trofile as reference assay: results from the OSCAR Study Group. New Microbiol 33: 195–206. 20954437

[19] Svicher V, Balestra E, Cento V, Sarmati L, Dori L, et al.. (2011) HIV-1 dual/mixed tropic isolates show different genetic and phenotypic characteristics and response to maraviroc in vitro. Antiviral Res 90: 42–53. S0166-3542(11)00033-7 [pii];10.1016/j.antiviral.2011.02.005 [doi]. 10.1016/j.antiviral.2011.02.005 21349294

[20] Swenson LC, Mo T, Dong WW, Zhong X, Woods CK, et al.. (2011) Deep sequencing to infer HIV-1 co-receptor usage: application to three clinical trials of maraviroc in treatment-experienced patients. J Infect Dis 203: 237–245. jiq030 [pii];10.1093/infdis/jiq030 [doi]. 10.1093/infdis/jiq030 PMC3071057 21288824

[21] Saliou A, Delobel P, Dubois M, Nicot F, Raymond S, et al.. (2011) Concordance between two phenotypic assays and ultradeep pyrosequencing for determining HIV-1 tropism. Antimicrob Agents Chemother 55: 2831–2836. AAC.00091-11 [pii];10.1128/AAC.00091-11 [doi]. 10.1128/AAC.00091-11 PMC3101380 21464245

[22] Raymond S, Delobel P, Mavigner M, Cazabat M, Souyris C, et al.. (2008) Correlation between genotypic predictions based on V3 sequences and phenotypic determination of HIV-1 tropism. AIDS 22: F11–F16. 10.1097/QAD.0b013e32830ebcd4 [doi]. 10.1097/QAD.0b013e32830ebcd4 18753930

[23] Boyd MT, Simpson GR, Cann AJ, Johnson MA, Weiss RA (1993) A single amino acid substitution in the V1 loop of human immunodeficiency virus type 1 gp120 alters cellular tropism. J Virol 67: 3649–3652. 8497073 10.1128/jvi.67.6.3649-3652.1993 PMC237718

[24] Dimonte S, Mercurio F, Svicher V, D’Arrigo R, Perno CF, et al.. (2011) Selected amino acid mutations in HIV-1 B subtype gp41 are associated with specific gp120v(3) signatures in the regulation of co-receptor usage. Retrovirology 8: 33. 1742-4690-8-33 [pii];10.1186/1742-4690-8-33 [doi]. 10.1186/1742-4690-8-33 PMC3117778 21569409

[25] Fouchier RA, Brouwer M, Broersen SM, Schuitemaker H (1995) Simple determination of human immunodeficiency virus type 1 syncytium-inducing V3 genotype by PCR. J Clin Microbiol 33: 906–911. 7790458 10.1128/jcm.33.4.906-911.1995 PMC228065

[26] Koito A, Stamatatos L, Cheng-Mayer C (1995) Small amino acid sequence changes within the V2 domain can affect the function of a T-cell line-tropic human immunodeficiency virus type 1 envelope gp120. Virology 206: 878–884. S0042-6822(85)71010-0 [pii];10.1006/viro.1995.1010 [doi]. 10.1006/viro.1995.1010 7856100

[27] Pastore C, Nedellec R, Ramos A, Pontow S, Ratner L, et al.. (2006) Human immunodeficiency virus type 1 coreceptor switching: V1/V2 gain-of-fitness mutations compensate for V3 loss-of-fitness mutations. J Virol 80: 750–758. 80/2/750 [pii];10.1128/JVI.80.2.750–758.2006 [doi]. 10.1128/JVI.80.2.750-758.2006 PMC1346864 16378977

[28] Swenson LC, Mo T, Dong WW, Zhong X, Woods CK, et al.. (2011) Deep V3 sequencing for HIV type 1 tropism in treatment-naive patients: a reanalysis of the MERIT trial of maraviroc. Clin Infect Dis 53: 732–742. cir493 [pii];10.1093/cid/cir493 [doi]. 10.1093/cid/cir493 21890778

