# Supplementary material for: The Genotypic False Positive Rate Determined by V3 Population Sequencing Can Predict the Burden of HIV-1 CXCR4-using Species Detected by Pyrosequencing
Source: PLoS One. 2013 Jan 14;8(1):e53603. doi: 10.1371/journal.pone.0053603 (PMC3544916; doi:10.1371/journal.pone.0053603)
Supplement: Text S1 — V3 population-sequencing. (DOC) [file pone.0053603.s001.doc]

**Text S1. V3 population-sequencing.**

The methodology for V3 population-sequencing is reported as follows. HIV-1 RNA was extracted from each isolate with a commercially available kit (QIAamp RNA-Viral-Mini-kit, Qiagen) (7). The V3 region of *env* gene was then reverse-transcribed and amplified by polymerase chain reactions (PCR) using the forward primer V3S2 5’-CAGCACAGTACAATGTACACA-3’ (nucleotide [nt]: 630-650 of HIV-1 HXB2 gp120 *env* gene) and the reverse primer V3AS5 5’-CTTCTCCAATTGTCCCTCA-3’ (nt: 1292-1310). The conditions for reverse transcription and amplification were: one cycle at 50°C for 30 min, one cycle 94°C for 2 min, 40 cycles (94°C 30s, 52°C 30s, 72°C 40s), and a final step at 72°C for 10 min, using the following reaction mix: 25 ul of RNA template, 8 ul of 5 mM Mg^2+^, 3 ul of Dnase Rnase free water, 0.75 ul of each primer at a concentration of 10 uM, 1 ul of Rnase out (40 U/ul), 1.5 ul of RT/Taq, 1 ul of dNTPs at a concentration of 10 mM for a total of 40 ul. When the RT-PCR product was not visible on agarose gel a semi-nested PCR was used with the inner forward primer V3S6 5’-CTGTTAAATGGCAGTCTAGC-3’ (nt: 682-702) and the reverse primer V3AS5. Amplification conditions were: one cycle at 93°C for 12 min, 40 cycles (95°C 30s, 52°C 30s, 72°C 40s) and a final step at 72°C for 10 min, using the following reaction mix: 5 ul of buffer taq 10X, 4 ul of Mg^2+^ at a concentration of 25 mM, 32.5 ul of Dnase Rnase free water, 0.9 ul of each primer at a concentration of 10 uM, 1 ul of dNTPs at a concentration of 10 mM, 0.7 ul of Taq (5 u/ul) for a total of 45 ul. The PCR product was purified by Microcon PCR purification kit (Millipore). Negative and positive control samples were included in each PCR run to exclude false-positive and false-negative reactions. PCR-products were then sequenced by using the BigDye terminator v.3.1 cycle sequencing kit (Applied-Biosystems), and an automated sequencer (ABI-3100). Four different overlapping sequence-specific primers were used to ensure the coverage of the V3-sequence by at least two sequence segments. The sequencing conditions were: one cycle 96°C 3 min, 25 cycles (96°C 30s, 50°C 10s, 60°C 4 min); the following primers were used: V3S6 5’-CTGTTAAATGGCAGTCTAGC-3’, V3S5 5’-GTTAAATGGCAGTCTAGCAG-3’, V3AS1 5’-GAAAAATTCCCCTCCACAATT-3’ and V3AS3bis 5’-CAATTTCTGGGTCCCCTC-3’. The sequences were analysed using SeqScape-v.2.5 software. The quality endpoint for each individual, was ensured by a coverage of the entire V3 sequence by at least two sequence segments. Translated protein sequences were generated and aligned using Bioedit 7.0 software and CLUSTALW1.8. To ensure the quality of the data, sequences were excluded from the analysis if a) contained stop codons; b) contained ambiguities consisting of >2 bases per nucleotide position.

V3-sequences are in process to be submitted to GenBank.
